# (*E*)-3-(4-Decyl­oxyphen­yl)-1-(2-hydroxy­phen­yl)prop-2-en-1-one

**DOI:** 10.1107/S1600536809010848

**Published:** 2009-03-28

**Authors:** Zainab Ngaini, Norashikin Irdawaty Abd Rahman, Hasnain Hussain, Ibrahim Abdul Razak, Hoong-Kun Fun

**Affiliations:** a Department of Chemistry, Faculty of Resource Science and Technology, Universiti Malaysia Sarawak, 94300 Kota Samarahan, Sarawak, Malaysia; b Department of Molecular Biology, Faculty of Resource Science and Technology, Universiti Malaysia Sarawak, 94300 Kota Samarahan, Sarawak, Malaysia; c X-ray Crystallography Unit, School of Physics, Universiti Sains Malaysia, 11800 USM, Penang, Malaysia

## Abstract

In the title compound, C_25_H_32_O_3_, the enone group is in an *s*–*cis* configuration. The dihedral angle between the benzene rings is 8.84 (7)°. An intra­molecular O—H⋯O inter­action between the keto and hydr­oxy groups forms an *S*(6) ring motif. Inter­molecular C—H⋯O inter­actions link the mol­ecules into supra­molecular chains along the *c* axis which are subsequently stacked down the *b* axis; the crystal structure is further consolidated by C—H⋯π inter­actions.

## Related literature

For general background, see: Bhat *et al.* (2005 ▶); Xue *et al.* (2004 ▶); Satyanarayana *et al.* (2004 ▶); Won *et al.* (2005 ▶); Zhao *et al.* (2005 ▶). For related structures, see: Ng, Razak *et al.* (2006 ▶); Ng, Patil *et al.* (2006 ▶); Razak *et al.* (2009 ▶); Ngaini *et al.* (2009 ▶). For details of hydrogen-bond motifs, see: Bernstein *et al.* (1995 ▶). For the stability of the temperature controller used in the data collection, see: Cosier & Glazer, 1986 ▶.
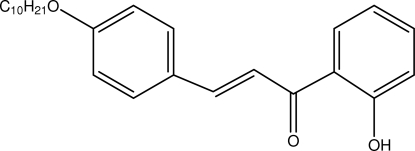


## Experimental

### 

#### Crystal data

C_25_H_32_O_3_
*M* *_r_* = 380.51
Monoclinic, 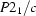

*a* = 21.2700 (4) Å
*b* = 7.6779 (2) Å
*c* = 13.2330 (3) Å
β = 101.720 (1)°
*V* = 2116.01 (8) Å^3^
*Z* = 4
Mo *K*α radiation
μ = 0.08 mm^−1^
*T* = 100 K
0.44 × 0.28 × 0.04 mm

#### Data collection

Bruker APEXII CCD area-detector diffractometer
Absorption correction: multi-scan (*SADABS*; Bruker, 2005 ▶) *T*_min_ = 0.967, *T*_max_ = 0.997
25687 measured reflections
6221 independent reflections
4014 reflections with *I* > 2σ(*I*)
*R*_int_ = 0.056

#### Refinement

*R*[*F* ^2^ > 2σ(*F* ^2^)] = 0.058
*wR*(*F* ^2^) = 0.165
*S* = 1.04
6221 reflections
258 parameters
H atoms treated by a mixture of independent and constrained refinement
Δρ_max_ = 0.37 e Å^−3^
Δρ_min_ = −0.26 e Å^−3^

### 

Data collection: *APEX2* (Bruker, 2005 ▶); cell refinement: *SAINT* (Bruker, 2005 ▶); data reduction: *SAINT*; program(s) used to solve structure: *SHELXTL* (Sheldrick, 2008 ▶); program(s) used to refine structure: *SHELXTL*; molecular graphics: *SHELXTL*; software used to prepare material for publication: *SHELXTL* and *PLATON* (Spek, 2009 ▶).

## Supplementary Material

Crystal structure: contains datablocks global, I. DOI: 10.1107/S1600536809010848/tk2402sup1.cif

Structure factors: contains datablocks I. DOI: 10.1107/S1600536809010848/tk2402Isup2.hkl

Additional supplementary materials:  crystallographic information; 3D view; checkCIF report

## Figures and Tables

**Table 1:** Hydrogen-bond geometry (Å, °)

*D*—H⋯*A*	*D*—H	H⋯*A*	*D*⋯*A*	*D*—H⋯*A*
O1—H1*O*1⋯O2	0.91 (2)	1.68 (2)	2.526 (2)	152 (2)
C15—H15*A*⋯O3^i^	0.93	2.48	3.406 (2)	174
C20—H20*B*⋯*Cg*1^ii^	0.97	2.85	3.702 (2)	147
C22—H22*A*⋯*Cg*1^iii^	0.97	2.84	3.712 (2)	149
C16—H16*A*⋯*Cg*2^iii^	0.97	2.87	3.596 (2)	132

Symmetry codes: (i) 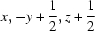; (ii) 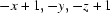; (iii) 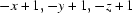. *Cg*1 and *Cg*2 are the centroids of the C1–C6 and C10–C15 rings, respectively.

**Fig. 1.**
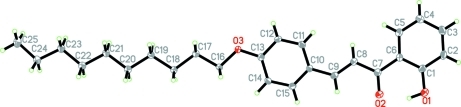
The molecular structure of (I), showing 50% probability displacement ellipsoids and the atom numbering scheme.

**Fig. 2.**
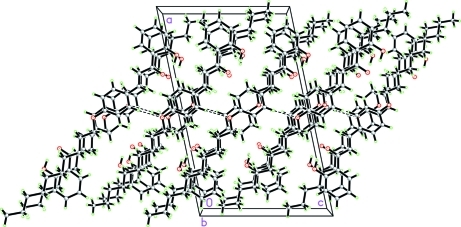
The crystal packing viewed down the b axis.

## supplementary crystallographic information

### Comment

Chalcone is one of the important intermediates in the biosynthesis of flavonoid. Chalcones derivatives are reported to exhibit biological properties such as an anti-malarial (Xue *et al.*, 2004), anti-cancer (Bhat *et al.*, 2005), anti-inflammatory (Won *et al.*, 2005), anti-platelet (Zhao *et al.*, 2005), and as well as anti-hyperglycemic (Satyanarayana *et al.*, 2004) activities.

Chalcone derivatives possessing alkyl chains of varying length have been synthesized in our laboratory. They were tested against *E. coli* ATCC 8739 for their anti-bacterial activities and showed anti-microbial activity. In this paper, we report the structure of one of the chalcone derivatives mentioned above.

In (I), Fig. 1, the enone group is in an *s*-*cis* configuration as indicated by the torsion angle O2—C7—C8—C9 of 1.2 (2)°. The least-square plane through the enone moiety makes dihedral angle of 3.64 (10)° with C1—C6 benzene ring whereas the dihedral angle formed with the C10—C15 benzene ring is 7.72 (10)°. The dihedral angle between these benzene rings is 8.84 (7)°. The alkoxyl group is co-planar with the attached benzene ring as shown by the torsion angle C16—O3—C13—C14 of -1.6 (2)°.

The strain induced by a short H5A···H8A contact (2.11 Å) leads to the slight opening of the C5—C6—C7 angle to 123.03 (13)°. Likewise, the widening of C8—C9—C10 (128.65 (14)°) and C9—C10—C11 (123.18 (13)°) angles are the result of a close H8A···H11A (2.32 Å) interatomic contact. These features were also observed in related structures reported previously (Ng, Razak *et al.*, 2006; Ng, Patil *et al.*, 2006; Razak *et al.*, 2009; Ngaini *et al.*, 2009). An intramolecular O1-H1O1···O2 interaction between the keto group and the hydroxy generates an *S*(6) ring motif (Bernstein *et al.*, 1995).

In the crystal structure, C15—H15A···O3 (*x*, -*y* + 1/2, *z* + 1/2) intermolecular interactions link the molecules into extended chains along the *c* axis (Table 1 and Fig. 2). These chains are subsequently stacked down the *b* axis. The crystal packing is further stabilized by the presence of C—H···π interactions formed between atoms C16, C20 and C22 in the alkoxyl tail and the benzene rings (Table 1).

### Experimental

A mixture of 2-hydroxyacetophenone (2.72 ml, 20 mmol), 4-decyloxybenzaldehyde (5.25 ml, 20 mmol) and KOH (4.04 g, 72 mmol) in methanol (60 ml) was heated at reflux for 10 h. The reaction mixture was cooled to room temperature and acidified with cold diluted HCl (2 N). The resulting precipitate was filtered, washed and dried. After redissolving in hexane and followed by few days of slow evaporation, crystals were collected.

### Refinement

All the C-bound H atoms were positioned geometrically and refined using a riding model with C—H = 0.93–0.97 Å. The *U*_iso_ values were constrained to be 1.5U_eq_(C) for methyl-H and 1.2U_eq_(C) for other H atoms. The rotating model group was applied for the methyl group. In the case of O1, the hydrogen atom was located from a difference Fourier map and refined without constraints.

### Crystal data

**Table:**

C_25_H_32_O_3_	*F*(000) = 824
*M**_r_* = 380.51	*D*_x_ = 1.194 Mg m^−^^3^
Monoclinic, *P*2_1_/*c*	Mo *K*α radiation, λ = 0.71073 Å
Hall symbol: -P 2ybc	Cell parameters from 3600 reflections
*a* = 21.2700 (4) Å	θ = 2.8–30.1°
*b* = 7.6779 (2) Å	µ = 0.08 mm^−^^1^
*c* = 13.2330 (3) Å	*T* = 100 K
β = 101.720 (1)°	Plate, yellow
*V* = 2116.01 (8) Å^3^	0.44 × 0.28 × 0.04 mm
*Z* = 4	

### Data collection

**Table:**

Bruker APEXII CCD area-detector diffractometer	6221 independent reflections
Radiation source: sealed tube	4014 reflections with *I* > 2σ(*I*)
graphite	*R*_int_ = 0.056
π and ω scans	θ_max_ = 30.1°, θ_min_ = 1.0°
Absorption correction: multi-scan (*SADABS*; Bruker, 2005)	*h* = −30→30
*T*_min_ = 0.967, *T*_max_ = 0.997	*k* = −10→10
25687 measured reflections	*l* = −18→18

### Refinement

**Table:**

Refinement on *F*^2^	Primary atom site location: structure-invariant direct methods
Least-squares matrix: full	Secondary atom site location: difference Fourier map
*R*[*F*^2^ > 2σ(*F*^2^)] = 0.058	Hydrogen site location: inferred from neighbouring sites
*wR*(*F*^2^) = 0.165	H atoms treated by a mixture of independent and constrained refinement
*S* = 1.04	*w* = 1/[σ^2^(*F*_o_^2^) + (0.085*P*)^2^] where *P* = (*F*_o_^2^ + 2*F*_c_^2^)/3
6221 reflections	(Δ/σ)_max_ < 0.001
258 parameters	Δρ_max_ = 0.37 e Å^−^^3^
0 restraints	Δρ_min_ = −0.26 e Å^−^^3^

### Special details

**Table:**

Experimental. The crystal was placed in the cold stream of an Oxford Cryosystems Cobra open-flow nitrogen cryostat (Cosier & Glazer, 1986) operating at 100.0 (1) K.
Geometry. All e.s.d.'s (except the e.s.d. in the dihedral angle between two l.s. planes) are estimated using the full covariance matrix. The cell e.s.d.'s are taken into account individually in the estimation of e.s.d.'s in distances, angles and torsion angles; correlations between e.s.d.'s in cell parameters are only used when they are defined by crystal symmetry. An approximate (isotropic) treatment of cell e.s.d.'s is used for estimating e.s.d.'s involving l.s. planes.
Refinement. Refinement of *F*^2^ against ALL reflections. The weighted *R*-factor *wR* and goodness of fit *S* are based on *F*^2^, conventional *R*-factors *R* are based on *F*, with *F* set to zero for negative *F*^2^. The threshold expression of *F*^2^ > σ(*F*^2^) is used only for calculating *R*-factors(gt) *etc*. and is not relevant to the choice of reflections for refinement. *R*-factors based on *F*^2^ are statistically about twice as large as those based on *F*, and *R*- factors based on ALL data will be even larger.

### Fractional atomic coordinates and isotropic or equivalent isotropic displacement parameters (Å^2^)

**Table:**

	*x*	*y*	*z*	*U*_iso_*/*U*_eq_	
O1	0.77177 (6)	−0.18603 (17)	1.07201 (8)	0.0262 (3)	
O2	0.67717 (5)	−0.08052 (16)	0.93853 (8)	0.0229 (3)	
O3	0.46966 (5)	0.31479 (15)	0.38134 (8)	0.0184 (3)	
C1	0.80643 (7)	−0.1716 (2)	0.99763 (11)	0.0190 (3)	
C2	0.87159 (7)	−0.2144 (2)	1.02373 (12)	0.0221 (4)	
H2A	0.8895	−0.2502	1.0905	0.026*	
C3	0.90932 (8)	−0.2037 (2)	0.95104 (13)	0.0238 (4)	
H3A	0.9527	−0.2317	0.9691	0.029*	
C4	0.88302 (7)	−0.1510 (2)	0.85025 (12)	0.0243 (4)	
H4A	0.9087	−0.1447	0.8012	0.029*	
C5	0.81885 (7)	−0.1087 (2)	0.82397 (12)	0.0202 (4)	
H5A	0.8015	−0.0748	0.7566	0.024*	
C6	0.77893 (7)	−0.1153 (2)	0.89626 (11)	0.0164 (3)	
C7	0.71034 (7)	−0.0670 (2)	0.87122 (11)	0.0168 (3)	
C8	0.68075 (7)	−0.0001 (2)	0.76851 (11)	0.0170 (3)	
H8A	0.7050	0.0083	0.7176	0.020*	
C9	0.61889 (7)	0.0488 (2)	0.74806 (11)	0.0162 (3)	
H9A	0.5973	0.0365	0.8021	0.019*	
C10	0.58106 (7)	0.1188 (2)	0.65252 (11)	0.0148 (3)	
C11	0.60383 (7)	0.1283 (2)	0.56004 (11)	0.0173 (3)	
H11A	0.6451	0.0898	0.5583	0.021*	
C12	0.56528 (7)	0.1943 (2)	0.47184 (11)	0.0181 (3)	
H12A	0.5806	0.1993	0.4109	0.022*	
C13	0.50347 (7)	0.2538 (2)	0.47327 (11)	0.0155 (3)	
C14	0.48019 (7)	0.2480 (2)	0.56425 (11)	0.0169 (3)	
H14A	0.4393	0.2891	0.5661	0.020*	
C15	0.51927 (7)	0.1794 (2)	0.65231 (11)	0.0178 (3)	
H15A	0.5037	0.1738	0.7130	0.021*	
C16	0.40477 (7)	0.3738 (2)	0.37618 (11)	0.0166 (3)	
H16A	0.4040	0.4690	0.4241	0.020*	
H16B	0.3784	0.2798	0.3935	0.020*	
C17	0.37994 (7)	0.4340 (2)	0.26649 (11)	0.0167 (3)	
H17A	0.3836	0.3389	0.2198	0.020*	
H17B	0.4068	0.5286	0.2512	0.020*	
C18	0.31030 (7)	0.4956 (2)	0.24639 (11)	0.0174 (3)	
H18A	0.3063	0.5922	0.2919	0.021*	
H18B	0.2830	0.4018	0.2613	0.021*	
C19	0.28817 (7)	0.5531 (2)	0.13439 (11)	0.0171 (3)	
H19A	0.3153	0.6484	0.1212	0.021*	
H19B	0.2946	0.4571	0.0899	0.021*	
C20	0.21838 (7)	0.6114 (2)	0.10436 (11)	0.0179 (3)	
H20A	0.2113	0.7086	0.1476	0.022*	
H20B	0.1906	0.5167	0.1161	0.022*	
C21	0.20115 (7)	0.6662 (2)	−0.00900 (11)	0.0197 (3)	
H21A	0.2293	0.7608	−0.0197	0.024*	
H21B	0.2095	0.5690	−0.0512	0.024*	
C22	0.13201 (7)	0.7246 (2)	−0.04652 (11)	0.0198 (3)	
H22A	0.1231	0.8218	−0.0048	0.024*	
H22B	0.1034	0.6300	−0.0375	0.024*	
C23	0.11847 (7)	0.7792 (2)	−0.15963 (12)	0.0210 (4)	
H23A	0.1467	0.8751	−0.1677	0.025*	
H23B	0.1289	0.6826	−0.2006	0.025*	
C24	0.04954 (7)	0.8345 (2)	−0.20226 (12)	0.0223 (4)	
H24A	0.0400	0.9375	−0.1657	0.027*	
H24B	0.0208	0.7424	−0.1899	0.027*	
C25	0.03726 (8)	0.8736 (3)	−0.31744 (13)	0.0296 (4)	
H25A	−0.0062	0.9124	−0.3402	0.044*	
H25B	0.0441	0.7700	−0.3544	0.044*	
H25C	0.0662	0.9630	−0.3304	0.044*	
H1O1	0.7313 (10)	−0.153 (3)	1.0406 (17)	0.053 (7)*	

### Atomic displacement parameters (Å^2^)

**Table:**

	*U*^11^	*U*^22^	*U*^33^	*U*^12^	*U*^13^	*U*^23^
O1	0.0252 (6)	0.0380 (8)	0.0154 (5)	0.0058 (6)	0.0043 (4)	0.0038 (5)
O2	0.0205 (6)	0.0322 (7)	0.0166 (5)	0.0022 (5)	0.0052 (4)	0.0040 (5)
O3	0.0157 (5)	0.0255 (7)	0.0137 (5)	0.0054 (5)	0.0025 (4)	0.0040 (4)
C1	0.0206 (8)	0.0206 (9)	0.0152 (7)	0.0009 (7)	0.0024 (6)	−0.0017 (6)
C2	0.0218 (8)	0.0255 (10)	0.0162 (7)	0.0055 (7)	−0.0025 (6)	0.0014 (6)
C3	0.0182 (8)	0.0260 (10)	0.0258 (8)	0.0050 (7)	0.0008 (6)	−0.0011 (7)
C4	0.0202 (8)	0.0302 (10)	0.0234 (8)	0.0027 (8)	0.0066 (6)	0.0020 (7)
C5	0.0207 (8)	0.0217 (9)	0.0178 (7)	0.0012 (7)	0.0027 (6)	0.0028 (6)
C6	0.0160 (7)	0.0162 (8)	0.0161 (7)	0.0003 (6)	0.0009 (5)	0.0002 (6)
C7	0.0180 (7)	0.0154 (8)	0.0164 (7)	−0.0009 (6)	0.0020 (5)	−0.0005 (6)
C8	0.0191 (7)	0.0172 (8)	0.0147 (7)	−0.0002 (7)	0.0033 (5)	0.0011 (6)
C9	0.0194 (7)	0.0155 (8)	0.0139 (7)	−0.0026 (6)	0.0039 (5)	−0.0023 (6)
C10	0.0151 (7)	0.0147 (8)	0.0140 (6)	−0.0009 (6)	0.0018 (5)	−0.0003 (6)
C11	0.0155 (7)	0.0187 (9)	0.0181 (7)	0.0020 (6)	0.0041 (5)	−0.0015 (6)
C12	0.0187 (7)	0.0221 (9)	0.0146 (7)	0.0018 (7)	0.0062 (5)	0.0011 (6)
C13	0.0169 (7)	0.0152 (8)	0.0136 (7)	0.0003 (6)	0.0016 (5)	−0.0003 (6)
C14	0.0135 (7)	0.0204 (9)	0.0170 (7)	0.0015 (6)	0.0034 (5)	−0.0016 (6)
C15	0.0167 (7)	0.0230 (9)	0.0144 (7)	−0.0017 (7)	0.0048 (5)	−0.0004 (6)
C16	0.0131 (7)	0.0211 (9)	0.0159 (7)	0.0018 (6)	0.0036 (5)	0.0008 (6)
C17	0.0165 (7)	0.0180 (8)	0.0154 (7)	0.0013 (6)	0.0024 (5)	0.0010 (6)
C18	0.0162 (7)	0.0207 (9)	0.0153 (7)	0.0017 (7)	0.0027 (5)	0.0016 (6)
C19	0.0161 (7)	0.0197 (9)	0.0154 (7)	0.0004 (6)	0.0030 (5)	0.0018 (6)
C20	0.0168 (7)	0.0190 (9)	0.0178 (7)	0.0006 (7)	0.0028 (5)	0.0007 (6)
C21	0.0179 (7)	0.0232 (9)	0.0175 (7)	0.0037 (7)	0.0023 (6)	0.0015 (6)
C22	0.0192 (7)	0.0213 (9)	0.0186 (7)	0.0018 (7)	0.0029 (6)	0.0009 (6)
C23	0.0194 (8)	0.0236 (9)	0.0191 (7)	0.0021 (7)	0.0016 (6)	0.0005 (6)
C24	0.0180 (7)	0.0244 (10)	0.0228 (8)	0.0023 (7)	0.0005 (6)	0.0014 (7)
C25	0.0279 (9)	0.0333 (11)	0.0238 (8)	0.0024 (8)	−0.0038 (7)	0.0034 (7)

### Geometric parameters (Å, °)

**Table:**

O1—C1	1.3488 (18)	C16—C17	1.5126 (19)
O1—H1O1	0.91 (2)	C16—H16A	0.9700
O2—C7	1.2479 (17)	C16—H16B	0.9700
O3—C13	1.3643 (17)	C17—C18	1.526 (2)
O3—C16	1.4411 (17)	C17—H17A	0.9700
C1—C2	1.398 (2)	C17—H17B	0.9700
C1—C6	1.418 (2)	C18—C19	1.5263 (19)
C2—C3	1.375 (2)	C18—H18A	0.9700
C2—H2A	0.9300	C18—H18B	0.9700
C3—C4	1.397 (2)	C19—C20	1.524 (2)
C3—H3A	0.9300	C19—H19A	0.9700
C4—C5	1.377 (2)	C19—H19B	0.9700
C4—H4A	0.9300	C20—C21	1.529 (2)
C5—C6	1.403 (2)	C20—H20A	0.9700
C5—H5A	0.9300	C20—H20B	0.9700
C6—C7	1.476 (2)	C21—C22	1.521 (2)
C7—C8	1.469 (2)	C21—H21A	0.9700
C8—C9	1.342 (2)	C21—H21B	0.9700
C8—H8A	0.9300	C22—C23	1.524 (2)
C9—C10	1.4563 (19)	C22—H22A	0.9700
C9—H9A	0.9300	C22—H22B	0.9700
C10—C15	1.394 (2)	C23—C24	1.520 (2)
C10—C11	1.406 (2)	C23—H23A	0.9700
C11—C12	1.379 (2)	C23—H23B	0.9700
C11—H11A	0.9300	C24—C25	1.523 (2)
C12—C13	1.395 (2)	C24—H24A	0.9700
C12—H12A	0.9300	C24—H24B	0.9700
C13—C14	1.393 (2)	C25—H25A	0.9600
C14—C15	1.390 (2)	C25—H25B	0.9600
C14—H14A	0.9300	C25—H25C	0.9600
C15—H15A	0.9300		
			
C1—O1—H1O1	104.5 (14)	C16—C17—H17A	108.9
C13—O3—C16	118.47 (11)	C18—C17—H17A	108.9
O1—C1—C2	117.51 (14)	C16—C17—H17B	108.9
O1—C1—C6	122.27 (14)	C18—C17—H17B	108.9
C2—C1—C6	120.21 (14)	H17A—C17—H17B	107.7
C3—C2—C1	120.24 (14)	C17—C18—C19	110.85 (12)
C3—C2—H2A	119.9	C17—C18—H18A	109.5
C1—C2—H2A	119.9	C19—C18—H18A	109.5
C2—C3—C4	120.52 (14)	C17—C18—H18B	109.5
C2—C3—H3A	119.7	C19—C18—H18B	109.5
C4—C3—H3A	119.7	H18A—C18—H18B	108.1
C5—C4—C3	119.56 (15)	C20—C19—C18	115.48 (12)
C5—C4—H4A	120.2	C20—C19—H19A	108.4
C3—C4—H4A	120.2	C18—C19—H19A	108.4
C4—C5—C6	121.68 (14)	C20—C19—H19B	108.4
C4—C5—H5A	119.2	C18—C19—H19B	108.4
C6—C5—H5A	119.2	H19A—C19—H19B	107.5
C5—C6—C1	117.76 (13)	C19—C20—C21	111.31 (12)
C5—C6—C7	123.03 (13)	C19—C20—H20A	109.4
C1—C6—C7	119.21 (13)	C21—C20—H20A	109.4
O2—C7—C8	119.42 (13)	C19—C20—H20B	109.4
O2—C7—C6	119.67 (13)	C21—C20—H20B	109.4
C8—C7—C6	120.90 (13)	H20A—C20—H20B	108.0
C9—C8—C7	120.17 (14)	C22—C21—C20	115.12 (12)
C9—C8—H8A	119.9	C22—C21—H21A	108.5
C7—C8—H8A	119.9	C20—C21—H21A	108.5
C8—C9—C10	128.65 (14)	C22—C21—H21B	108.5
C8—C9—H9A	115.7	C20—C21—H21B	108.5
C10—C9—H9A	115.7	H21A—C21—H21B	107.5
C15—C10—C11	118.18 (13)	C21—C22—C23	112.27 (12)
C15—C10—C9	118.64 (13)	C21—C22—H22A	109.2
C11—C10—C9	123.18 (13)	C23—C22—H22A	109.2
C12—C11—C10	120.31 (14)	C21—C22—H22B	109.2
C12—C11—H11A	119.8	C23—C22—H22B	109.2
C10—C11—H11A	119.8	H22A—C22—H22B	107.9
C11—C12—C13	120.56 (13)	C24—C23—C22	114.63 (13)
C11—C12—H12A	119.7	C24—C23—H23A	108.6
C13—C12—H12A	119.7	C22—C23—H23A	108.6
O3—C13—C14	124.49 (13)	C24—C23—H23B	108.6
O3—C13—C12	115.33 (12)	C22—C23—H23B	108.6
C14—C13—C12	120.18 (13)	H23A—C23—H23B	107.6
C15—C14—C13	118.67 (14)	C23—C24—C25	112.51 (14)
C15—C14—H14A	120.7	C23—C24—H24A	109.1
C13—C14—H14A	120.7	C25—C24—H24A	109.1
C14—C15—C10	122.08 (14)	C23—C24—H24B	109.1
C14—C15—H15A	119.0	C25—C24—H24B	109.1
C10—C15—H15A	119.0	H24A—C24—H24B	107.8
O3—C16—C17	106.62 (11)	C24—C25—H25A	109.5
O3—C16—H16A	110.4	C24—C25—H25B	109.5
C17—C16—H16A	110.4	H25A—C25—H25B	109.5
O3—C16—H16B	110.4	C24—C25—H25C	109.5
C17—C16—H16B	110.4	H25A—C25—H25C	109.5
H16A—C16—H16B	108.6	H25B—C25—H25C	109.5
C16—C17—C18	113.54 (12)		
			
O1—C1—C2—C3	179.46 (16)	C9—C10—C11—C12	179.31 (15)
C6—C1—C2—C3	−0.6 (3)	C10—C11—C12—C13	0.5 (2)
C1—C2—C3—C4	−0.4 (3)	C16—O3—C13—C14	−1.6 (2)
C2—C3—C4—C5	0.4 (3)	C16—O3—C13—C12	178.28 (13)
C3—C4—C5—C6	0.6 (3)	C11—C12—C13—O3	−179.46 (14)
C4—C5—C6—C1	−1.5 (2)	C11—C12—C13—C14	0.4 (2)
C4—C5—C6—C7	178.57 (16)	O3—C13—C14—C15	178.78 (14)
O1—C1—C6—C5	−178.55 (15)	C12—C13—C14—C15	−1.1 (2)
C2—C1—C6—C5	1.5 (2)	C13—C14—C15—C10	0.9 (2)
O1—C1—C6—C7	1.4 (2)	C11—C10—C15—C14	0.0 (2)
C2—C1—C6—C7	−178.58 (15)	C9—C10—C15—C14	−179.99 (14)
C5—C6—C7—O2	178.04 (16)	C13—O3—C16—C17	179.70 (13)
C1—C6—C7—O2	−1.9 (2)	O3—C16—C17—C18	178.01 (13)
C5—C6—C7—C8	−3.0 (2)	C16—C17—C18—C19	−179.58 (13)
C1—C6—C7—C8	177.06 (15)	C17—C18—C19—C20	177.93 (14)
O2—C7—C8—C9	1.2 (2)	C18—C19—C20—C21	179.96 (14)
C6—C7—C8—C9	−177.73 (15)	C19—C20—C21—C22	179.28 (14)
C7—C8—C9—C10	179.58 (15)	C20—C21—C22—C23	179.36 (14)
C8—C9—C10—C15	−172.82 (16)	C21—C22—C23—C24	178.50 (14)
C8—C9—C10—C11	7.2 (3)	C22—C23—C24—C25	−175.39 (15)
C15—C10—C11—C12	−0.7 (2)		

### Hydrogen-bond geometry (Å, °)

**Table:**

*D*—H···*A*	*D*—H	H···*A*	*D*···*A*	*D*—H···*A*
O1—H1O1···O2	0.91 (2)	1.68 (2)	2.526 (2)	152 (2)
C15—H15A···O3^i^	0.93	2.48	3.406 (2)	174
C20—H20B···Cg1^ii^	0.97	2.85	3.702 (2)	147
C22—H22A···Cg1^iii^	0.97	2.84	3.712 (2)	149
C16—H16A···Cg2^iii^	0.97	2.87	3.596 (2)	132

Symmetry codes: (i) *x*, −*y*+1/2, *z*+1/2; (ii) −*x*+1, −*y*, −*z*+1; (iii) −*x*+1, −*y*+1, −*z*+1.
